# Supplementary material for: Crucial Role of Ni Point Defects and Sb Doping for Tailoring the Thermoelectric Properties of ZrNiSn Half-Heusler Alloy: An Ab Initio Study
Source: Materials (Basel). 2024 Feb 25;17(5):1061. doi: 10.3390/ma17051061 (PMC10935130; doi:10.3390/ma17051061)
Supplement: Supplementary file 1 [file materials-17-01061-s001.zip › materials-2813992-supplementary.pdf]

Crucial Role of Ni-points Defects and Sb-doping in Tailoring Thermoelectric Properties of ZrNiSn Half-Heusler Alloy: an Ab Initio Study.

E. Ascrizzi,[1] C. Ribaldone,[1] and S. Casassa*[1]
Department of Chemistry, University of Torino, via Giuria 5, 10125, Torino, Italy
(*Electronic mail: silvia.casassa@unito.it)

(Dated: 24 February 2024)

S1. SUPPORTING INFORMATION

A. ZrNiSn primitive cell

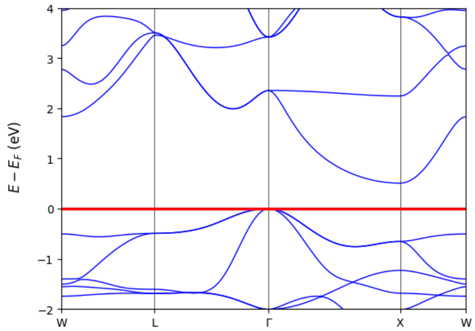

FIG. S1. ZrNiSn primitive cell (3 atoms) band structure. It is visible the indirect band gap.

B. TiNiSn

The TiNiSn lattice parameter of the unit cell is 5.20 Å at PBE level. In Fig. S2, the band structure and the density of states of a 96 atoms supercell are reported.

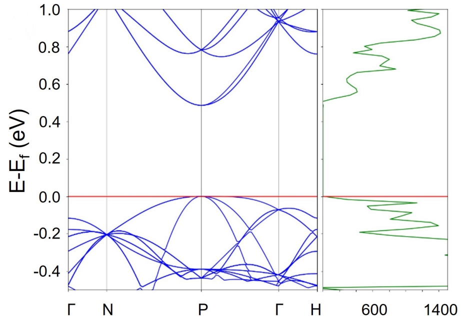

FIG. S2. Band structure and density of states for TiNiSn $2\times2\times2$ supercell.

	a	B_0	c_{11}	c_{12}	c_{44}	ν	Y
This work	5.95	199.6	395.0	102.0	113.3	0.24	311.5
Exp.	6.11[1]	124.6[2]	224.8[2]	74.6[2]	75.1[2]	0.249[2]	187.7[2]

TABLE S1. Lattice parameter a in Å. Bulk modulus, B_0, elastic constants, c_{11}, c_{12}, c_{44}, Young's modulus, Y, in GPa and Poisson ratio, ν, for *SC96-P*. Experimental values, measured at room temperature, are also reported.

C. ZrNiSn structural properties

S2. THERMOELECTRIC PROPERTIES

Computed data for $SC96-P$ at 300 K are compared with literature results in the Table S2. Electron conductivity values are higher than that reported in any of the experimental studies considered. This could be due to the heavy approximations of our computational model, above all the frozen band approximation and the constant relaxation time approximation.

S3. EXPERIMENTAL DATA FROM XIE ET AL.[6]

In the Table below we report the total thermal conductivity measured for these samples at different temperatures that we have used in the estimation of the ZT for the different structures.

S4. BASIS SET FOR THE METALLIC HALF-HEUSLER

The basis used in the calculation of the full-Heusler alloy are the original ones available on the CRYSTAL basis set database (www.crystal.unito.it/basis_set.html) In particular: Zr: L. Valenzano et al.[12] Sn: J. Laun, et al.[13] Ni: M.D. Towler et al.[14]

[1] J. Schmitt, Z. M. Gibbs, G. J. Snyder, and C. Felser, "Resolving the true band gap of ZrNiSn half-Heusler thermoelectric materials," Mater. Horiz. **2**, 68–75 (2015).
[2] G. Rogl, A. Grytsiv, M. Gürth, A. Tavassoli, C. Ebner, A. Wünschek, S. Puchegger, V. Soprunyuk, W. Schranz, E. Bauer, H. Müller, M. Zehetbauer, and P. Rogl, "Mechanical properties of half-Heusler alloys," Acta Mater. **107**, 178–195 (2016).
[3] H. Muta, T. Kanemitsu, K. Kurosaki, and S. Yamanaka, "High-temperature thermoelectric properties of Nb-doped MNiSn (M=Ti, Zr) half-Heusler compound," J. Alloys Compd. **469**, 50–55 (2009).

Study	n_C $[10^{19}\mathrm{cm}^{-3}]$	S $[\mu\mathrm{V/K}]$	PF $[10^{-4}\mathrm{WK}^{-2}\mathrm{m}^{-1}]$	σ $[10^4\Omega^{-1}m^{-1}]$	k_e $[\mathrm{Wm}^{-1}\mathrm{K}^{-1}]$	ZT
This work	0.3	-313	14	1.5	0.51	0.05
Ref[1], (EXP)	0.25	-325		0.1		
Ref[3], (EXP)		-350	10			
Ref[4], (EXP)		-340		1		
Ref[5], (THEO)		-505		40	0.01	
This work	3.6	-114	24	19	1.9	0.06
Ref[6], (EXP)	5.0	-225		3		0.06
Ref[7], (EXP)	4.0	-250		1		
Ref[8], (EXP)		-125		3		
Ref[9], (EXP)		-125		2	0.25	0.02
Ref[10], (THEO)		-228		1.9	0.46	
Ref[11], (THEO)		-125		8	1	

TABLE S2. Carrier concentration, n_C, Seebeck coefficient, S, power factor, PF, electron conductivity, σ, electron thermal conductivity, k_e and figure of merit, ZT for ZrNiSn Half-Heusler alloy, *SC96-P*, at 300 K. The higher part of the table collects data from works in which n_C is lower and S decreases with temperature, while the lower part of the table collects data from work in which n_C is higher and S increases with temperature. For the ZT calculation, k_{tot} has been taken from the experimental work of Xie et al.[6], see also Table S3.

[4]H. Miyazaki, T. Nakano, M. Inukai, K. Soda, Y. Izumi, T. Muro, J. Kim, M. Takata, M. Matsunami, S. Kimura, and *et al.*, "Electronic and Local Crystal Structures of the ZrNiSn Half-Heusler Thermoelectric Material," Mater. Trans. **55**, 1209–1214 (2014).

[5]N. Kumar, H. S. Saini, Nisha, M. Singh, and M. K. Kashyap, "Enhanced thermoelectric properties of Ta-doped Half-Heusler ZrNiSn," Mater. Today:. Proc. **26**, 3478–3481 (2020).

[6]H. Xie, H. Wang, C. Fu, Y. Liu, G. J. Snyder, X. Zhao, and T. Zhu, "The intrinsic disorder related alloy scattering in ZrNiSn half-Heusler thermoelectric materials," Sci. Rep. **4**, 1–6 (2014).

[7]M. Schrade, K. Berland, A. Kosinskiy, J. P. Heremans, and T. G. Finstad, "Shallow impurity band in ZrNiSn," J. Appl. Phys. **127**, 045103 (2020).

[8]Q. Shen, L. Chen, T. Goto, T. Hirai, J. Yang, G. P. Meisner, and C. Uher, "Effects of partial substitution of Ni by Pd on the thermoelectric properties of ZrNiSn-based half-Heusler compounds," Appl. Phys. Lett. **79**, 4165–4167 (2001).

[9]X. Liu, J. He, H. Xie, X. Zhao, and T. Zhu, "Fabrication and thermoelectric properties of Yb-doped ZrNiSn half-Heusler alloys," International Journal of Smart and Nano Materials **3**, 64–71 (2012).

[10]S. Yousuf, T. M. Bhat, S. Singh, Z. Saleem, S. A. Mir, S. A. Khandy, A. Q. Seh, S. A. Sofi, M. Nabi, V. K. Sharma, and D. C. Gupta, "Applicability of semi-classical Boltzmann transport theory in understanding the thermoelectric properties of ZrNiSn and ZrNiPb half-heuslers," AIP Conf. Proc. **2115**, 030420 (2019).

[11]S. S. Shastri and S. K. Pandey, "Thermoelectric properties, efficiency and thermal expansion of ZrNiSn half-Heusler by first-principles calculations," J. Phys.: Condens. Matter **32**, 355705 (2020).

[12]L. Valenzano, B. Civalleri, S. Chavan, S. Bordiga, M. H. Nilsen, S. Jakobsen, K. P. Lillerud, and C. Lamberti, "Disclosing the Complex Structure of UiO-66 Metal Organic Framework: A Synergic Combination of Experiment and Theory," Chem. Mater. **23**, 1700–1718 (2011).

[13]J. Laun, D. V. Oliveira, and T. Bredow, "Consistent gaussian basis sets of double- and triple-zeta valence with polarization quality of the fifth period for solid-state calculations," J. Comput. Chem. **39**, 1285–1290 (2018), 29468714.

[14]M. D. Towler, N. L. Allan, N. M. Harrison, V. R. Saunders, W. C. Mackrodt, and E. Aprà, "Ab initio study of MnO and NiO," Phys. Rev. B **50**, 5041–5054 (1994).

T [K]	ZrNiSn	$ZrNiSn_{0.97}Sb_{0.03}$	$ZrNiSn_{0.94}Sb_{0.06}$
300	6	9.0	10.6
400	5.8	8.8	10.4
500	5.4	8.3	10.2
600	5.2	7.9	9.8
700	5.1	7.6	9.5
800	5.1	7.2	9.1
900	5.2	6.9	8.8

TABLE S3. Data from literature: measured values of k_{tot} in $[\mathrm{Wm}^{-1}\mathrm{K}^{-1}]$ by Xie et al.[6].
